# Cyberbullying Victimisation as a Mediator Between Social Media Use and Emotional Problems Among Elementary School Students

**DOI:** 10.3390/healthcare14020271

**Published:** 2026-01-21

**Authors:** Sanja Radić Bursać, Sabina Mandić, Martina Lotar Rihtarić, Dora Dodig Hundrić, Neven Ricijaš

**Affiliations:** 1 Teaching and Clinical Centre, University of Zagreb Faculty of Education and Rehabilitation Sciences, 10000 Zagreb, Croatia; sanja.radic.bursac@erf.unizg.hr; 2 Department of Behavioural Disorders, University of Zagreb Faculty of Education and Rehabilitation Sciences, 10000 Zagreb, Croatia; dora.dodig@erf.unizg.hr (D.D.H.); neven.ricijas@erf.unizg.hr (N.R.); 3 Department of Criminology, University of Zagreb Faculty of Education and Rehabilitation Sciences, 10000 Zagreb, Croatia; martina.lotar.rihtaric@erf.unizg.hr

**Keywords:** elementary schools’ students, social media use, emotional problems, emotional well-being, cyberbullying victimisation, mediating effects

## Abstract

**Background/Objectives:** Adolescence is a developmental period characterised by intensive use of social media and an increased prevalence of emotional problems such as depression and anxiety. Scientific evidence indicates that the modality of social media use (active or passive) can significantly predict these problems, with active use being linked to a higher likelihood of cyberbullying victimisation. As victimisation is associated with more severe emotional problems, social media represents an important context for understanding adolescent mental health. Following this, the main aim of this study was to examine how the modality of social media use (SMU) is related to emotional problems, and whether cyberbullying victimisation mediates this relationship. **Methods:** This study was conducted on a convenient sample of *N* = 1822 students (49.0% boys, 51.0% girls; M_age_ = 13.22 years, SD_age_ = 0.629) from a total of 64 elementary schools throughout Croatia. A modified Croatian version of the Active and Passive Use of Social Networks Scale, the Anxiety and Depression subscales of the Depression, Anxiety, Stress Scale—Youth Version, and the Cyber-Victimisation subscale of the European Cyberbullying Intervention Project Questionnaire were used. **Results:** The results indicate that passive SMU among boys is directly related only to anxiety, while that among girls contributes only to the explanation of depression. Regarding cyberbullying victimisation as a mediator, full mediation in the association between active SMU and emotional problems was found for both girls and boys. **Conclusions**: This represents a significant theoretical contribution, as well as a contribution to the development of psychosocial interventions.

## 1. Introduction

Adolescence is a developmental period that partially overlaps with the onset of puberty, typically around ages 10–12, and ends around 18 years of age, although recent findings indicate that adolescence may continue beyond this age [1,2,3]. Alongside both physical and psychological changes, this period is characterised by increased significance of peers [3,4], identity exploration [1,5], and experimentation with various (often risky) behaviours [6,7,8]. Adolescence is also a time when individuals begin to use digital technologies more intensively, acquire their first mobile devices, and create profiles on social media [9,10,11]. Some studies indicate a trend towards acquiring first mobile phones at increasingly younger ages [12,13], and that younger adolescents are the most intensive users of digital devices, spending up to 8 h a day in front of a screen, with at least 3 h on social media [10,14,15].

Adolescence is also a period during which an increasing number of mental health problems have been recorded in recent years, especially emotional problems, such as anxiety and depression. World Health Organisation highlights these issues, along with self-harm, as “*leading causes of disease burden for adolescents worldwide*” [16]. Globally, research demonstrates a prevalence of approximately 25–35% for depression and 20–30% for anxiety symptoms, depending on the geographic area [14,17,18,19,20,21]. Research in Croatia shows similar trends, with a significant increase in emotional problems among high school students in recent years [22,23,24]. For example, the results of a recent study by Mandić et al. [25] on a representative sample of *N* = 825 high school students in the City of Zagreb indicate the prevalence of serious symptoms of anxiety and depression, at 32.7% and 22.2%, respectively. It should be noted that these prevalence estimates are based on self-reported symptoms in population-based samples and do not represent clinically diagnosed psychiatric disorders.

Guided by studies reporting a very high frequency of social media use (hereafter: SMU) and a high prevalence of emotional problems among adolescents, more intensive exploration of the connection between these two constructs has been conducted in recent years. SMU is often cited as one of the predictors of emotional problems in young people, together with other individual and environmental factors such as personality traits, screen exposure, peer relationships, and experiences of stressful or traumatic events [13].

Regarding the SMU as a predictor, studies have been conducted in several directions. The most frequently studied aspect is the contribution of the frequency of SMU to the development of emotional problems. For example, various studies suggest a significantly higher presence of anxiety and depressive symptoms in those individuals who use social media more intensively/frequently on a daily basis [14,25,26]. Although data on the frequency of SMU are important, the results of some meta-analyses and longitudinal studies [27,28,29] show that only a small portion of the development of emotional problems can be explained by frequency of use (approximately 1–5% of the explained variance). This suggests that other aspects may be significant in explaining the development of emotional problems in young people. Following this, underlying motivation, activity and engagement on social media, exposure to different (often inappropriate) content, experiences of cyberbullying (both perpetration and victimisation), and symptoms indicating problematic and excessive SMU also appear to be important [30,31,32,33].

Furthermore, according to recent research, the extent of individuals’ engagement on social media can help explain effects on mental health [34,35,36]. As a result, increasing attention has been given to active and passive SMU. Active use involves direct interaction and communication with others, posting materials or statuses, commenting, liking, following, and messaging. In contrast, passive use refers to more “observational” activities, such as scrolling through feeds and observing others’ interactions without direct participation; information or posts are typically consumed without communicating with the content creator [35,37,38].

Following the above, different ways of SMU have different effects on general well-being and the potential development of emotional problems, as explained by the active–Passive Model of Social Media Use [38]. According to this model, active use tends to enhance social connectedness, feelings of belonging, and (emotional) well-being, whereas passive use is (often directly) associated with increased depressive and anxiety symptoms, and it leads to negative self-image [35,38]. In other words, this model suggests a negative correlation between passive use and emotional well-being, while active use has more positive (or neutral) effects on an individual’s emotional problems and well-being. The nature of this connection has been established in several other studies [36,39,40], but it should be noted that it is rarely direct, especially when it comes to active use. Rather, the data indicate that some factors lead to this connection, suggesting a potential mediating effects.

Regarding the passive SMU, research generally shows a direct association with emotional problems [36,39]. It is also important to highlight the gender differences identified in the study by Frison and Eggermont [39], which found a direct connection between passive use and depressive symptoms only in girls. However, there are some studies that have identified factors that fully or partially mediate that association. In a study by Burnell et al. [41] on a sample of *N* = 717 undergraduate students, fear of missing out (hereafter: FoMO) and social comparison were identified as mediators. Specifically, passive SMU had a significant positive effect on social comparison, which was positively correlated with FoMO; in turn, FoMO had a significant positive effect on depressive symptoms. These findings suggest that social comparison and FoMO play a significant role in the link between passive SMU and depressive symptoms [41]. In addition, envy [42], body (dis)satisfaction, and self-objectification [43] are also mentioned as significant mediators between passive SMU and emotional problems.

When it comes to active SMU, research predominately focuses on studying mediators in the context of “positive” mental health outcomes. For example, Frison and Eggermont [39] state that more active SMU leads to an increase in perceived online social support, which then leads to a reduction in depressive symptoms. This study is among the few that incorporate gender differences into the model. The results showed that the relationship between active SMU and depressed mood was significantly mediated by perceived online social support only for girls, while for boys, this relationship was not significant [39]. Only a small portion of studies have tested mediator effects between active SMU and the presence of emotional problems. One such study is that of Mao et al. [44], where in a sample of almost 500 undergraduate students, they found that active SMU was associated with loneliness through the mediation pathways of interpersonal satisfaction and FoMO. Another such study was conducted by Tang et al. [43], which, on a sample of over 2000 high school students, determined that self-objectification and body satisfaction are mediators between active SMU and depression symptoms. The referenced study also confirmed a significant serial mediation of self-objectification and body satisfaction in the relationship between active SMU and depression.

Following the literature review, it is evident that there are different factors that mediate the relationship between SMU (passive or active) and emotional problems (depression and anxiety) among adolescents. However, a research gap exists regarding cyberbullying victimisation as a potential mediator. Studies indicate that only active SMU is related to a greater risk of cyberbullying victimisation [45,46,47]. This association is explained by the greater online exposure of active users, increased opportunities to encounter potential perpetrators, higher levels of self-disclosure and public visibility [48,49], weaker ties with an audience that may misuse shared content or materials [50,51], and problematic SMU, which includes excessive use, poorer coping strategies, and risky interactions [51,52,53]. On the other hand, numerous studies show that adolescents who have been victims of cyberbullying experience more mental health problems, such as depression, anxiety, and suicidal thoughts [47,53,54,55,56]. Given the high prevalence of cyberbullying among adolescents, the established correlation between active SMU and the risk of cyber-victimisation, and consistent evidence of its negative effects on emotional well-being, a logical question arises regarding the possible mediating effect among these variables. Only a few studies have examined that effect, and one of them is a study by Sampasa-Kanyinga and Hamilton [57] involving over 5000 participants aged 11 to 20 years. Results imply that cyberbullying victimisation fully mediates the relationship between SMU and psychological distress (symptoms of anxiety and depression), such that more intensive use of social media is associated with an increased risk of cyberbullying victimisation, which in turn is associated with a higher risk of mental health problems. Although conducted with a sample of *N* = 502 adults, a more recent study by Barragan et al. [58] suggests that the relationship between SMU and depression and anxiety is robustly mediated by increased experiences of cyberbullying victimisation. The nature of the established association follows the same pattern as in the study conducted by Sampasa-Kanyinga and Hamilton [57]. Barragan et al. [58] included gender as a potential moderator of the indirect effect in the model and found no differences; the mediation effect of cyberbullying victimisation is the same for both genders. Although these studies confirm a mediating effect of cyberbullying victimisation between the frequency of SMU and problematic use and emotional problems, they do not specifically examine active and passive use of social media.

### Current Study

As a result of all the above, the importance of how young people use social media (active or passive use) in relation to their mental health and the development of emotional problems is indisputable. When it comes to the latter, in this study, emotional problems are conceptualised as risk indicators rather than clinically defined psychiatric disorders, consistent with a dimensional approach in non-clinical populations. This study contributes to the body of evidence on these important topics in several ways.

First, almost all studies available to the authors focus on the use of one or several social media platforms (mainly Facebook or Instagram) and are primarily focused on frequency of use or problematic use (excessive use or addiction), although it is known that the type of use is much more important. Second, regarding participant samples, most studies focus on high school or university students or adults, while the younger population, particularly elementary school students, is almost entirely neglected. Given the high prevalence of SMU, cyberbullying victimisation, and emotional problems recorded in this population, this study includes this understudied group.

Furthermore, there is a lack of research examining the direct and indirect effects between the social media use modality (active/passive) and emotional problems in general. Regarding cyberbullying victimisation as a potential mediator, even fewer studies are available. Those that have been conducted (e.g., [57,58]) focus on problematic or excessive SMU, while, to the author’s knowledge, those related to specific ways of use do not exist. It is also important to emphasise the need to include both modalities (active and passive) of SMU in the same model, as was done in this research.

In line with the Active–Passive Model of Social Media Use [38] and studies confirming a positive association between passive SMU and emotional problems [36,39], according to the first hypothesis, we assume that passive social media use has a significant positive direct effect on anxiety and depression (H1).

Given findings indicating that those who actively use social media are at greater risk of cyberbullying victimisation [45,46,47], according to the second hypothesis, we assume that cyberbullying victimisation mediates the relationship between active social media use and emotional problems. Specifically, more frequent active social media use is expected to be associated with more frequent cyberbullying victimisation, which in turn is linked to more symptoms of anxiety and depression (H2).

Furthermore, gender differences have been largely overlooked in most studies, with only two including gender in the model as a potential moderator of the indirect effect [39,58]. Research also shows that girls tend to use social media more frequently and report more pronounced emotional problems [25,59]. Based on these results, a stronger association between passive SMU and emotional problems can also be expected. Therefore, according to the third hypothesis, we expect significant differences in the parameter estimates of the tested model between boys and girls, such that among girls, passive SMU contributes more to the explanation of anxiety and depression (H3).

While being aware of the fact that previous longitudinal research does not provide unambiguous results in this field [29,60,61], the empirical foundation for our hypothesised model is based on previous longitudinal studies that have confirmed a direct relationship between screen time and SMU with adverse mental health outcomes among adolescents over time [62,63,64,65], as well as studies that have failed to support the reverse causal direction, using a longitudinal design [65,66]. Regarding the mediating role of victimisation, the direct association between passive SMU and emotional problems is theoretically sound, as passive SMU does not presume exposure on social media (posting pictures, videos, comments, etc.), thereby precluding some of the conditions for peer victimisation. Concurrently, although some existing literature has employed experiences of harassment and bullying as mediating variables between SMU and emotional outcomes [55,67,68], there remains a lack of empirical scrutiny of the distinctions between active and passive SMU. Testing for gender differences in the proposed model is also supported by previous longitudinal studies that confirmed more adverse negative emotional consequences among girls [69,70].

**Figure 1:**
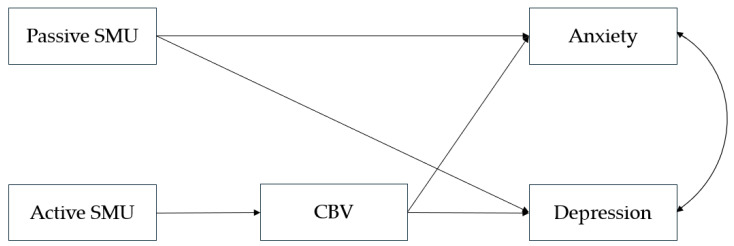
Hypothesised model of the relationships between different modalities of social media use, cyberbullying victimisation, and adolescents’ emotional problems.

The model based on H1 and H2 is presented in Figure 1.

## 2. Materials and Methods

### 2.1. Participants

This study was conducted on a convenient sample of *N* = 1876 students (7th and 8th grade) from a total of 64 elementary schools throughout Croatia. Of the initial sample, 55 participants did not report their gender and were excluded from further analyses. The final sample for analysis consisted of *N* = 1822 participants, of whom 49.0% (*n* = 892) were boys and 51.0% (*n* = 930) were girls. The participants’ ages ranged from 12 to 15 years (M_age_ = 13.22 years; SD_age_ = 0.629). Accordingly, 42.3% of the sample was from smaller (up to 10,000 inhabitants), 37.4% from medium (up to 100,000 inhabitants), and 20.3% from larger cities/municipalities (over 100,000 inhabitants).

### 2.2. Measures

For this study, a modified *Croatian version of the Active and Passive Use of Social Networks Scale* ([71], adapted from the original Passive and Active Facebook Use Measure (PAUM); [72]) was used. The scale measures two dimensions of social media use—active and passive—and consists of 10 items, which participants rate on a 5-point Likert scale (1—never; 5—very often). Five items measure active use (e.g., “*Browsing the newsfeed actively (liking and commenting on posts, pictures and updates).*”), and five items measure passive use of social networks (e.g., “*Checking to see what someone is up to.*”). Both dimensions of the modified scale demonstrate an appropriate level of internal consistency. Cronbach’s α coefficient for the active use dimension is 0.75, while that for the passive use dimension is 0.81 [71]. The internal consistency in this study is satisfactory for the active use subscale (α = 0.707, ω = 0.716), while the passive use subscale has internal consistency below 0.70 (α = 0.651, ω = 0.685). For this reason, an item-level analysis was conducted, which showed that the item “Browsing the newsfeed passively (without liking or commenting on anything)” had very low correlations with the other four items measuring passive social media use. Moreover, removing this item increased internal consistency; therefore, it was excluded from further analyses. A confirmatory factor analysis (CFA) of the resulting scale was conducted, comprising five items for active social media use and four items for passive social media use. The model fit indices indicated an acceptable fit to the data (χ^2^(26) = 239, *p* < 0.01, CFI = 0.942, RMSEA = 0.066, SRMR = 0.034). Factor loadings ranged from 0.49 to 0.67 for the active social media use dimension and from 0.48 to 0.65 for the passive social media use dimension. The internal consistency of the passive social media use dimension, measured with four items, was α = 0.719 and ω = 0.729.

To assess the severity of emotional problems, the Anxiety and Depression subscales of the *Depression, Anxiety, Stress Scale—Youth Version* (DASS-Y; [73]) was used. This self-report questionnaire consists of 7 items per subscale rated on a four-point scale (from 0—“not true for me” to 3—“very true for me”). The total score for each dimension is calculated by summing the response values for all items within the relevant subscale. Higher scores indicate more pronounced symptoms, that is, a greater prevalence of emotional problems [73]. The instrument’s authors reported high reliability (Depression: α = 0.89, ω = 0.90; Anxiety: α = 0.84, ω = 0.84) and good validity in a validation study [73]. The internal consistency of the subscales in this study was high (Depression: α = 0.915, ω = 0.916; Anxiety: α = 0.864, ω = 0.865).

To measure cyberbullying victimisation, the Cyber-Victimisation subscale of the *European Cyberbullying Intervention Project Questionnaire* (ECIPQ; [74]) was used. This subscale consists of 11 items (e.g., “*Someone hacked into my account and pretended to be me*.”) with five response options (0—never, 1—once or twice, 2—once a month, 3—once a week, 4—more times a week), where students indicate how often they experienced described behaviours in the past two months. Previous studies have found adequate internal consistency; for example, Del Rey et al. [75] report α = 0.97, while Álvarez-Marín et al. [76] and Herrera-López et al. [77] report ω = 0.82 to ω = 0.94 for this subscale in samples of elementary school students. The internal consistency of the mentioned subscale among the participants in this study is also satisfactory (α = 0.836; ω = 0.849).

In addition to the above, information on gender, age, grade level, and place of residence was also collected.

### 2.3. Procedure and Compliance with Ethical Principles

The study protocol was reviewed and approved by the Ethics Committee of the University of Zagreb Faculty of Education and Rehabilitation Sciences, as well as by the Croatian Ministry of Science and Education (with a positive opinion from the Education and Teacher Training Agency). The research was conducted as part of the implementation of the preventive programme “Tools for Modern Times”. Before the research began, written consent from parents was obtained. The research took place during the 2023/2024 school year, within the students’ regular classes, using the “pencil–paper” principle. Before participating, students were informed about the main aim of the study, how to complete the questionnaire, anonymity, and voluntary participation, and their right to withdraw from completing the questionnaire at any time.

### 2.4. Data Analysis

Statistical analyses were conducted using Jamovi 2.6.44 software. Descriptive statistics and Pearson correlations were calculated for all variables included in the tested model. The distributions of scores on all measures were examined. If they were normally distributed, differences were tested using a *t*-test. If distributions deviated significantly from normality according to Kim’s [78] criteria, differences between boys and girls were tested using the Mann–Whitney U test.

Research hypotheses were tested using a path analysis, while parameter estimates were calculated using the maximum likelihood method. Overall model fit was assessed using χ^2^ statistics, comparative fit index (CFI), root mean squared error (RMSEA), and standardised root mean square residual (SRMR). To estimate the model, the Hu and Bentler [79] guidelines for cut-off values were used. The bootstrapping method was used to examine whether cyberbullying victimisation mediates the relationship between active social media use and emotional problems (anxiety and depression). To determine whether there is a significant difference in the parameter estimates of the tested model between boys and girls, a multigroup path analysis was conducted. We tested whether constraining the regression coefficients to be equal across groups would lead to a significant improvement in model fit. If constraining the regression coefficients does not result in a significant improvement in model fit (i.e., the difference in χ^2^ is not significant at the 0.05 level), it will be concluded that there is a significant difference between the regression coefficients for boys and girls.

## 3. Results

Table 1 presents the descriptive statistics for all measures for boys and girls. The distribution of cyberbullying victimisation scores significantly deviates from normal among boys, while the distribution of anxiety scores significantly deviates among girls. Therefore, gender differences on these measures were tested using the Mann–Whitney U test.

From the results presented, we see that girls score significantly higher on all measures. Girls use social media more frequently in both passive and active modalities, experience cyberbullying more often, and report more pronounced symptoms of anxiety and depression.

Table 2 presents the correlations among variables for boys and girls. The results show that the correlations in both subsamples are similar. The positive correlation between the two modalities of SMU is expected, as students who use social media generally do so across both modalities, with fewer using it in only one way. Cyberbullying victimisation is positively correlated with both passive and active SMU and is also positively associated with students’ emotional problems.

Before hypothesis testing, gender measurement invariance was examined for the measures used in this study. Because the χ^2^ statistic becomes increasingly sensitive to deviations from the null hypothesis as sample size increases, large overall samples can lead to an inflated likelihood of rejecting measurement invariance. Therefore, changes in alternative fit indices are often preferred, as they are less affected by sample size [80]. For studies with sufficient statistical power, balanced group sizes, and partial invariance, Chen [81] recommended a cut-off of −0.010 for changes in CFI, along with thresholds of 0.015 for RMSEA and 0.030 for SRMR when testing metric invariance, or 0.015 for SRMR when evaluating scalar or residual invariance. Given the substantial sample size in the present study (*N* = 1872), the assessment of measurement invariance followed the criteria outlined by Chen [81]. When reporting differences in model fit indices, a negative value indicates a decrease, and a positive value indicates an increase, in fit for the more restrictive model relative to the less restrictive one. Testing metric invariance for active and passive social media use showed that the scale did not meet the criteria for configural invariance (ΔCFI = −0.028, ΔRMSEA = 0.016, ΔSRMR = 0.012). The Cyber-Victimisation measure demonstrated configural invariance (ΔCFI = −0.006, ΔRMSEA = 0.004, ΔSRMR = 0.007); however, for metric invariance, the decrease in CFI exceeded the criterion proposed by Chen [81] (ΔCFI = −0.031, ΔRMSEA = 0.001, ΔSRMR = 0.002). For the anxiety and depression measures, the results indicated configural invariance (ΔCFI = −0.003, ΔRMSEA = −0.002, ΔSRMR = 0.003 for anxiety, ΔCFI = −0.003, ΔRMSEA = 0.002, ΔSRMR = 0.001 for depression), metric invariance (ΔCFI = 0.000, ΔRMSEA = −0.006, ΔSRMR = 0.005 for anxiety, ΔCFI = −0.001, ΔRMSEA = −0.009, ΔSRMR = 0.009 for depression), and scalar invariance (ΔCFI = −0.002, ΔRMSEA = −0.003, ΔSRMR = 0.002 for anxiety, ΔCFI = −0.001, ΔRMSEA = −0.006, ΔSRMR = 0.002 for depression). For residual invariance, the change in CFI did not support invariance, whereas the changes in RMSEA and SRMR did (ΔCFI = −0.023, ΔRMSEA = 0.012, ΔSRMR = 0.13 for anxiety, ΔCFI = −0.011, ΔRMSEA = 0.004, ΔSRMR = 0.007 for depression). Therefore, residual invariance for anxiety and depression was not supported.

**Figure 2:**
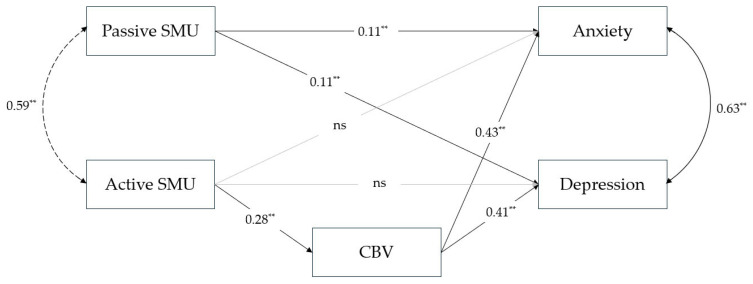
Path model of relation between different modalities of SMU, cyberbullying victimisation, and emotional problems (*N* = 1822). Note. Standardised parameters estimates are shown. ** *p* < 0.01. ns—non significant.

The tested model, presented in Figure 2., shows a good fit to the data (χ^2^(1) = 6.92; *p* < 0.01; CFI = 0.997; RMSEA = 0.058; SRMR = 0.013). Because this model has a small number of degrees of freedom, it should be noted that RMSEA is not an appropriate indicator of model fit, as it includes degrees of freedom in the denominator of its formula and therefore penalises complex models. This problem exists even with large samples (e.g., *N* = 1000) [82]. The results reported by Shi et al. [82] indicate that for samples larger than *N* = 200, SRMR and CFI can be used even for very small models (e.g., df = 2). Therefore, emphasis should be placed on SRMR and CFI, and it can be concluded that the model shows a very good fit.

Path analysis indicates a significant direct effect of passive SMU on anxiety *β* = 0.11, *b* = 0.556, 95% CI [0.291, 0.794] and depression *β* = 0.11, *b* = 0.578, 95% CI [0.287, 0.852] (Figure 2). We can conclude that the first hypothesis has been confirmed. There is a full mediating effect of cyberbullying victimisation on the association between active SMU and both aspects of emotional problems. The indirect effect of active SMU on both anxiety and depression via cyberbullying victimisation is *β* = 0.12, *b* = 0.674, 95% CI [0.518, 0.837] and *β* = 0.12, *b* = 0.698, 95% CI [0.544, 0.865], respectively. The second hypothesis has also been fully confirmed. The model explained 7.91% of the observed variance in cyberbullying victimisation, 23.06% of the observed variance in anxiety, and 19.38% of the observed variance in depression.

**Figure 3:**
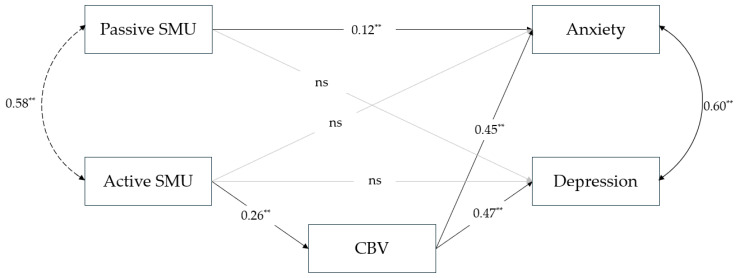
Multigroup path model of relation between different modalities of SMU, cyberbullying victimisation, and emotional problems for boys (*n* = 892). Note. Standardised parameters estimates are shown. ** *p* < 0.01. ns—non significant.

**Figure 4:**
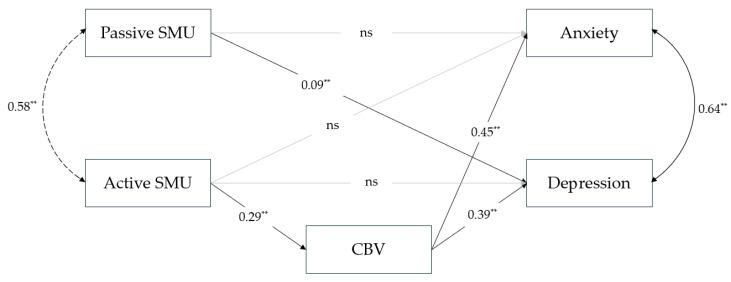
Multigroup path model of relation between different modalities of SMU, cyberbullying victimisation, and emotional problems for girls (*n* = 930). Note. Standardised parameters estimates are shown. ** *p* < 0.01. ns—non significant.

To examine the final hypothesis, which assumes significant differences in the parameter estimates of the tested model between boys and girls, a multigroup path analysis was conducted by gender. Given the model’s low degrees of freedom (df = 2), CFI and SRMR were considered, and it can be concluded that the multigroup model presented in Figure 3 and Figure 4 also shows a good fit to the data (*χ*^2^(2) = 6.67; *p* < 0.05; CFI = 0.997; RMSEA = 0.052; SRMR = 0.013). The difference in *χ*^2^ between first tested model and the multigroup model is statistically significant (*p* < 0.01), and the multigroup model provides a significantly better fit to the data.

Among boys (Figure 3), passive SMU had a significant direct effect on anxiety, *β* = 0.12, *b* = 0.478, 95% CI [0.138, 0.787] only. The effects of active SMU on emotional problems were indirect, through cyberbullying victimisation. The indirect effect of active SMU on anxiety through cyberbullying victimisation was *β* = 0.12, *b* = 0.536, 95% CI [0.349, 0.727], and the indirect effect on depression was *β* = 0.12, *b* = 0.663, 95% CI [0.447, 0.891]. The model explained 6.81% of the variance in cyberbullying victimisation, 21.36% of the variance in anxiety, and 21.94% of the variance in depression. Among girls (Figure 4), passive SMU had a significant direct effect on depression, *β* = 0.09, *b* = 0.563, 95% CI [0.144, 1.014] only. The effects of active SMU on emotional problems were indirect, mediated by cyberbullying victimisation. The indirect effect of active SMU on anxiety through cyberbullying victimisation was *β* = 0.13, *b* = 0.795, 95% CI [0.577, 1.051], and the indirect effect on depression was *β* = 0.11, *b* = 0.720, 95% CI [0.512, 0.973]. For the girls, the model explained 8.28% of the variance in cyberbullying victimisation, 23.86% of the variance in anxiety, and 16.98% of the variance in depression.

In the next step, we examined whether there was a significant difference in the parameter estimates for boys and girls by testing the model fit with the regression coefficients constrained. The multigroup model with constrained regression coefficients also shows a good fit to the data, *χ*^2^(9) = 35.60, *p* < 0.01, CFI = 0.985, RMSEA = 0.058, SRMR = 0.037. However, the *χ*^2^ value for the unconstrained multigroup model indicated a significantly better fit than the constrained model (*p* < 0.01). We therefore conclude that the regression coefficients differ significantly between boys and girls. Despite the significant differences in the regression coefficients, we cannot confirm the third hypothesis of this study because the differences in the coefficients do not align with the predicted direction. We expected direct effects of SMU on anxiety and depression to be more pronounced in girls than in boys, but in girls, passive SMU predicts only depression, not anxiety, whereas in boys, only the direct effect of passive SMU on anxiety is significant. We can conclude that the effects of passive SMU are not more pronounced in girls; rather, they represent different effects on emotional problems.

## 4. Discussion

The main goal of this study was to explore how the modality of SMU, i.e., whether adolescents use them actively or passively, is related to emotional problems, specifically anxiety and depression, and whether cyberbullying victimisation mediates this relationship.

Firstly, the results confirm that girls use social media more frequently than boys, both actively and passively. Additionally, girls show more symptoms of anxiety and depression. These findings are consistent with previous studies on adolescent populations, which have consistently reported similar trends among girls [25,59]. Regarding cyberbullying victimisation, this study indicates significant differences, with girls being victims more often than boys, although the effect size is low (r = −0.11). These results also align with recent meta-analyses and umbrella reviews, which, when analysing studies on adolescent samples, found significant gender differences, but with small effects and variations depending on age, geographic location, and similar factors [47,83]. Following often-inconsistent results, Lee et al. [84] state that research on cyberbullying victimisation should also consider different developmental perspectives depending on gender. They confirmed these differences through a longitudinal study across five waves with a sample of *N* = 3617 students aged 13 to 18. The main difference is that for most girls, cyberbullying victimisation decreases with age (with the largest decrease occurring after the age of 15), while for boys, victimisation levels peak between the ages of 13 and 15, and for a smaller proportion of boys, victimisation gradually increases after the age of 15 [84]. This is not surprising, given the established knowledge that boys mature somewhat later than girls, which may also explain these differences [2]. In other words, when interpreting gender differences in cyberbullying victimisation, it is necessary to consider the specificities of each developmental period within adolescence to avoid biased results.

Consistent with the first hypothesis, the results of testing the mediation model on the full sample, including both boys and girls, show significant direct positive effects between passive SMU and both anxiety and depression. In other words, the first hypothesis (H1) is fully confirmed. This connection was also established in previous studies, which unequivocally support the positive association between passive SMU and emotional problems (i.e., depression and anxiety) in adolescents (e.g., [36,39]). This association can be explained by the fact that those who passively observe the lives of their peers tend to make upward comparisons, i.e., comparing their own lives with those of peers they perceive as much better off [39], which can consequently lead to the development of emotional problems. However, to gain a deeper understanding of this relationship, future studies should further examine relevant underlying mechanisms and verify possible mediating effects, such as FoMO, envy, social comparison, and satisfaction with one’s own appearance.

Building on previous research that separately examined the effects of active SMU on cyberbullying victimisation [45,46,47], as well as the effects of victimisation on youth mental health [47,53,54,55,56], this study integrates these constructs into a single model, addressing a gap in the literature. In other words, in the second hypothesis, we proposed that cyberbullying victimisation mediates the relationship between active SMU and emotional problems. Specifically, more frequent active SMU is expected to be associated with more frequent cyberbullying victimisation, which in turn is linked to increased symptoms of anxiety and depression. Based on the prior studies presented in this paper, we conclude that the second hypothesis (H2) is fully supported, as cyberbullying victimisation fully mediates the association between active SMU and both aspects of emotional problems. Although causality cannot be inferred, this model suggests that children who actively use SMU have an increased likelihood of cyberbullying victimisation, and higher cyberbullying victimisation predicts symptoms of anxiety and depression. As there is no research exploring the mediating effect of victimisation in the relationship between active SMU and emotional problems, and therefore direct comparisons of the results obtained are impossible, such findings are not surprising when we consider other research in this area. Firstly, it is known that young people who are more active on social media and more frequently share content from their own lives are at greater risk of having these materials misused and of becoming victims compared to those who only passively scroll through online content and do not provide the opportunity to misuse shared materials. Furthermore, the fact that someone has been a victim of any unacceptable behaviour, whether in a real or digital environment, inevitably affects their mental well-being and the presence of emotional problems, especially during adolescence (e.g., [47,55,85]). As these results are based on a cross-sectional design, further longitudinal studies are needed to test the proposed mediation pathway and to establish the temporal order and causal direction among the variables.

The identified research gap concerning gender differences served as the basis for setting up the third hypothesis, where we expected significant differences in the parameter estimates of the tested model between boys and girls, such that among girls, passive SMU would contribute more to explaining anxiety and depression. A significant difference was found between the first model (tested on the entire sample) and the multigroup model (separated by gender), with the multigroup model providing a better fit to the data. This suggests that the model should be considered separately by gender, which could be overlooked if the analysis is conducted on the entire sample. When cyberbullying victimisation was modelled as a mediator, the indirect associations between active social media use and emotional problems (both depression and anxiety) were statistically significant for both girls and boys, indicating a pattern consistent with full mediation. However, the relationship between passive SMU and emotional problems in relation to gender is noteworthy. Although it was assumed that the direct effect of passive SMU on both anxiety and depression would be significant for both genders, but stronger for girls, this was not confirmed, leading to the conclusion that the third hypothesis (H3) was not supported. The results of this study indicate that passive SMU among boys is directly related only to anxiety, and not to depression at all (Figure 3). A possible explanation may lie in the different factors that contribute to anxiety among boys compared to girls. Society places distinct expectations on young men, who are encouraged to pursue different goals, which in turn leads to differences in anxiety-related stimuli. Boys are taught to restrain fear and insecurity, to solve problems, achieve objectives, and are encouraged to be successful, whereas girls are permitted to express fears and worries and are encouraged to be passive and compliant [86]. It is therefore not surprising that the predominant sources of anxiety among men are work-related, whereas for women, they are tied to difficulties in interpersonal relationships [87]. It is possible that, during passive SMU, boys focus primarily on the performance, status, and achievement of others, making these the key elements on which they base social comparison. As content shared on social media predominantly showcases success, boys may consequently experience fear of their own potential failure. Conversely, girls, whose primary source of anxiety arises from challenges in close relationships, develop and maintain such relationships both on social media and in real-life contexts [88,89], making it less likely that they rely predominantly on social media information when evaluating these relationships. However, longitudinal research is necessary to examine this proposed explanation.

In contrast, among girls, passive SMU contributes only to the explanation of depression, with no significant connection to anxiety (Figure 4). Although they studied only depression, Frison and Eggermont [39] reached the same conclusions in their research that passive use is directly related to depression only in girls, while the connection was not significant in boys. Thorisdottir et al. [36] also found that passive use is more strongly related to symptoms of depressive mood among girls. Possible (at least partial) explanations for this direction of results can be found in studies exploring gender differences in the characteristics of SMU and the underlying mechanisms that lead to the presence of emotional problems. For example, in the study by Nesi and Prinstein [90], results suggested that the association between technology-based social comparison and feedback-seeking, and depressive symptoms was particularly strong for girls compared to boys. These results are explained by the fact that girls are more inclined to publish their own photos more frequently, to compare their physical appearance and attractiveness with others in the online world, and perceive such comparisons as more self-relevant, which, in turn, makes them more threatening to self-worth. Furthermore, based on established interpersonal theories of depression, which show that the link between interpersonal stressors and depressive symptoms may be particularly strong for girls (Rudolph, 2002, as cited in [90]), and guided by the fact that technology-based social comparison and feedback-seeking is a type of stressor for girls, these behaviours are likely to be associated with depressive symptoms. This perspective is consistent with prior empirical findings indicating that females more frequently use social media for social comparison and experience greater vulnerability to body image concerns, both of which are associated with depressive symptoms (e.g., [91,92,93]).

At this point, it is also important to note that the anxiety and depression measures demonstrated scalar invariance, whereas the cyber-victimisation measure showed only configural invariance, and the active and passive social media use measure did not exhibit gender invariance at any level. Therefore, the observed gender differences should be interpreted with caution because they may, at least in part, reflect the lack of the highest level of measurement invariance for the instruments used in this study.

Although our study may provide some further insights into the relationship between active and passive SMU and adolescents’ emotional well-being, there are some limitations that should be addressed. First, it is a cross-sectional study, which precludes causal inferences about the relationships, so the observed associations should also be examined with longitudinal design. Second, the measures used in this study did not demonstrate strict invariance. Therefore, as noted earlier, gender differences should be interpreted with caution. Third, it would also be necessary to include a wider age range, for example, from approximately 11 to 18 years, a period that encompasses a broader span of adolescence. From a developmental perspective, it is possible that certain behaviours, such as experiencing cyberbullying victimisation [84], may occur at different stages in the lives of girls and boys during adolescence, which also emphasises the need for longitudinal research. Future studies would benefit from integrating family-context variables with social media use and peer-related processes to provide a more comprehensive developmental model.

Despite the mentioned limitations, the results of this study have important methodological and practical implications. First, this study underscores the importance of examining both the direct and indirect effects of SMU modality on emotional problems among adolescents. Furthermore, the results demonstrate and reaffirm that this subject must be considered from a gender perspective, due to identified gender differences in the tested mediation model, differences in SMU, the presence and severity of mental health problems, the underlying mechanisms of SMU, and their various roles in cyberbullying. Cyberbullying victimisation, as an insufficiently studied mediator in the association between the modality of SMU and the development of emotional problems, should be explored more fully to gain insight into its effects. These results may also have significant practical implications. The finding that active SMU is related to cyberbullying victimisation, which in turn is related to emotional problems, while passive SMU has direct effects on emotional problems may be useful for developing psychosocial interventions. By focusing on each modality of use, such interventions could potentially help prevent both victimisation and emotional problems.

## 5. Conclusions

The way adolescents use social media, whether actively or passively, is one of the most significant factors in explaining the development of emotional problems, along with cyberbullying victimisation, which is highly prevalent among adolescents. In this study, we advanced existing scientific knowledge by demonstrating the importance of cyberbullying victimisation in the relationship between active SMU and emotional problems. The results of this study indicate that passive SMU among boys is directly related only to anxiety, and not to depression at all, while among girls, passive use contributes only to the explanation of depression, with no significant connection to anxiety. Regarding cyberbullying victimisation as a mediator, full mediation in the association between active SMU and emotional problems was found for both girls and boys. This represents a significant theoretical contribution, as well as a contribution to the development of psychosocial interventions, where emphasis should be placed not only on the time spent online or the specific social media used, but also on the way in which it is used and the underlying mechanisms, which can lead to mental health problems in young people.

## Figures and Tables

**Table 1:** Descriptive statistics for active and passive social network use, cyberbullying victimisation, anxiety, and depressiveness (*N_boys_* = 892; *N_girls_* = 930).

Variable	Gender	M	SD	Min	Max	Skewness	Kurtosis	*t*-Test/Mann–Whitney U	Effect Size
1. Passive SMU	Boys	2.98	0.877	1	5	−0.11	−0.38	*t* = −9.48 **	d = −0.45
	Girls	3.38	0.890	1	5	−0.26	−0.37		
2. Active SMU	Boys	2.21	0.779	1	5	0.53	−0.10	*t* = −8.14 **	d = −0.38
	Girls	2.52	0.829	1	5	0.32	−0.32		
3. CBV	Boys	3.52	5.356	0	37	2.68	9.01	Z = −4.15 **	r_rb_ = −0.11
	Girls	4.22	5.436	0	32	2.04	4.55		
4. Anxiety	Boys	2.79	3.587	0	21	1.85	3.75	Z = −9.81 **	r_rb_ = −0.27
	Girls	5.00	5.436	0	21	2.04	4.55		
5. Depression	Boys	2.76	4.249	0	21	2.12	4.41	*t* = −7.81 **	d = −0.37
	Girls	4.55	5.383	0	21	1.21	0.51		

Note. SMU—social media use; CBV—cyberbullying victimisation; Min—the lowest obtained score; Max—the highest obtained score; ** *p* < 0.01.

**Table 2:** Pearson coefficients between variables for boys and girls (*N_boys_* = 892; *N_girls_* = 930).

Variable	1	2	3	4	5
1. Passive SMU	-	0.57 **	0.21 **	0.18 *	0.14 **
2. Active SMU	0.58 **	-	0.26 **	0.13 **	0.11 **
3. CBV	0.21 **	0.29 **	-	0.46 **	0.47 **
4. Anxiety	0.19 **	0.22 **	0.48 **	-	0.69 **
5. Depression	0.17 **	0.17 **	0.41 **	0.70 **	-

Note. SMU—social media use; CBV—cyberbullying victimisation; Correlation coefficients below the diagonal are for girls and coefficients above the diagonal are for boys. * *p* < 0.05; ** *p* < 0.01.

## Data Availability

The raw data supporting the conclusions of this article will be made available by the authors on request. The data are not publicly available because they are part of an ongoing research project.
